# Psychometric validation study of the Korean version of the Functional Assessment of Cancer Therapy-Vanderbilt Cystectomy Index

**DOI:** 10.1371/journal.pone.0190570

**Published:** 2018-01-05

**Authors:** Myong Kim, Seung-June Oh, Cheol Kwak, Hyeon Hoe Kim, Ja Hyeon Ku

**Affiliations:** 1 Department of Urology, Asan Medical Center, University of Ulsan College of Medicine, Seoul, Republic of Korea; 2 Department of Urology, Seoul National University Hospital, Seoul National University College of Medicine, Seoul, Republic of Korea; Aligarh Muslim University, INDIA

## Abstract

**Purposes:**

To evaluate the reliability and validity of a Korean version of the Functional Assessment of Cancer Therapy (FACT)-Vanderbilt Cystectomy Index (VCI) in Korean patients who underwent radical cystectomy (RC) and urinary diversion (UD).

**Materials and methods:**

We prospectively recruited 108 RC and UD patients who did not have evidence of recurrence from 1994 December to 2015 March. All participants were instructed to complete the Korean FACT-VCI and Short-Form 36-Item Health Survey (SF-36; 1^st^ measurement) and to repeat the Korean FACT-VCI survey one month later (2^nd^ measurement). Statistical analysis included intraclass correlation, Cronbach’s α, time and UD type fixed mixed linear model, principal components analysis, and criterion-related validity with SF-36.

**Results:**

Korean FACT-VCI was internally consistent (α = 0.802) and had adequate test-retest reliability (interclass correlation = 0.803 and 0.822). The three components model of principal component analysis (cumulative explanatory power, 49.2%) confirmed the internal structural validity of the additional concerns (AC) component of the Korean FACT-VCI, and each component represented the “voiding problem”, “bowel problem”, and “social/functional problem with equivalent explanatory power (19.5%, 15.4%, and 14.4%). Korean FACT-VCI domain scores were generally well correlated with SF-36 domain scores (Pearson correlation coefficients range: 0.286–0.688; all p <0.01). Mixed linear models revealed that the major effect of measurement times was not significant on FACT-VCI (p = 0.589)

**Conclusions:**

This prospective study confirms the reliability and validation of the Korean FACT-VCI. We expect that this validated tool can be widely utilized in the health-related quality of life studies of Korean patients.

## Introduction

Radical cystectomy (RC) and urinary diversion (UD) remains the gold standard treatment for invasive bladder cancer [1,2]. But, RC and UD for bladder cancer is one of the most traumatic cancer surgeries causing psychological stress and alternation in life-style. Significant morbidities include sexual dysfunction, voiding problem, or change in body image [3]. Furthermore, extension of RC indications in high risk T1 diseases [1,2] and increasing long-term survival have increased the impact of the treatment on health-related quality of life (HRQOL).

Several studies have addressed HRQOL following RC [4–8]. However, the tools used to assess HRQOL were instruments for general disease [4,8] or other malignancies [7], or lacked external validation[5]. To address these shortcomings, Cookson et al. developed a novel questionnaire for patients receiving RC and UD. The questionnaire—the Functional Assessment of Cancer Therapy (FACT)-Vanderbilt Cystectomy Index (VCI)–is based on the FACT-General (FACT-G) questionnaire, whose reliability and validity have been confirmed [9], in addition to items for concerning distressing symptoms following RC [10]. The reliability and validity of the original version of FACT-VCI were also confirmed [10], and psychometric validation has been confirmed utilizing externally extended cohorts [11]. This validated questionnaire is now generally accepted as a credible tool for assessment of HRQOL in patients who have undergone RC and UD, and has been adopted in many HRQOL studies of bladder cancer [12,13].

In the process of translation of a questionnaire written in one language into a version in another language, the translated versions must reflect differences in the culture and custom of subjects who use a given language. In a first step toward obtaining a validated assessment tool for HRQOL of patients who underwent RC and UD in Korea, we previously performed the translation and linguistic validation of the Korean version of the FACT-VCI (Korean FACT-VCI), which consists of 27-items from the FACT-G component, and additional 17-items regarding RC-related concerns (FACT-AC component) [14]. As the next step, we prospectively performed the psychometric validation of Korean FACT-VCI to establish its reliability and validity in Korean populations.

## Matrials and methods

### Ethical statements

This prospective study was approved by the institutional review board (IRB) of Seoul National University Hospital (Approval No. H-1307-127-508), Seoul, Republic of Korea. All participants voluntarily agreed to participate in this study and signed a written informed consent form which was approved by our IRB. All personal information was anonymized before analysis.

### Establishment of Korean version of FACT-VCI questionnaire

The original English version of the FACT-VCI consists of 27-items derived from the FACT-G (FACT-G component), which is divided into physical (GP), social/family (GS), emotional (GE), and functional well-being (GF) domains [9], in addition to 17-items regarding RC and UD-related concerns (FACT-AC component) that are specifically related to urinary, bowel, and sexual function [10]. We previously performed the translation and linguistic validation of Korean FACT-VCI [14]. Briefly, translation and linguistic validation were carried out according to permission for translation, forward translation, reconciliation, backward translation, cognitive debriefing, and final proof-reading [14].

### Study populations

Population size was determined by referral to the previous proposed optimal subject-to-item ratios for internal component validation, which were suggested as being between 5:1 and 10:1 [15,16]. Patients who underwent RC and UC at our institution from 1994 December to 2015 March were prospectively recruited at least one month before the evaluation. Evidence of disease recurrence, illiteracy, history of psychical disorder, and withholding of consent to participate were exclusion criteria. All patients were instructed to complete the Korean FACT-VCI survey and the Korean version of the Short-Form 36-Item Health Survey (SF-36) version 2.0 on the first visit (1^st^ measurement) [17,18], and to complete the Korean FACT-VCI in one month later (2^nd^ measurement). Basic demographic and disease-related variable were also prospectively collected.

### Reliability assessment

Test-retest reliability of the Korean FACT-VCI including 17-items of the FACT-AC component were determined by intraclass correlation coefficients with 95% confidence intervals (CIs), using samples of patients who completed the 1^st^ and 2^nd^ measurements. Cronbach’s α was estimated to assess whether the 17-items of the FACT-AC component had a value >0.70, which is considered indicative of internal consistency, using samples of persons who completed the 1^st^ measurement [19]. A linear mixed model with a fixed measurement time and urinary diversion type was utilized to confirm the absolute agreement between repeated tests of the 17-items of the FACT-AC component.

### Validity assessment

Principal components analysis was performed to determine the internal compositional validity of 16 gender-neutral items (item BL3, able to have erection, was excluded a male-only item) of the FACT-AC component. The varimax criterion was adopted for analytic rotation in this factor analysis [20]. Following a previous study [11] and recommendations [21,22], item loading ≥0.30 was considered as substantive in each component. External validity was confirmed by criterion-related association between the Korean FACT-VCI including the 17-items of the FACT-AC component and SF-36 version 2.0. Pearson’s correlation coefficients with p-values were estimated for that purpose [23]. All statistical analyses were performed using commercially available software (SPSS^®^ version 21.0, Chicago, IL, United States).

## Results

### Demographics and clinical data

The study included 108 patients who met the inclusion criteria and who completed the 1^st^ measurement (FACT-VCI and SF-36 questionnaires) at median of 7.3 months (interquartile range [IQR], 1.4–35.4) postoperatively. Median age at time of surgery was 66.8 years (IQR, 58.1–71.9). Of the 108 patients, 95 (88.0%) were male, 52 (48.1%) underwent orthotopic neobladder (ONB) and 56 (51.9%) received ileal conduit (IC) as UD. Among the recruited subjects, 14 (13.0%) could not complete the 2^nd^ measurement (repeated FACT-VCI).

### Reliability assessment

Table 1 presents the data for the Korean FACT-VCI item variance with its test-retest reliability and internal consistency. Patients showed the full range of responses (0–4), which varied according to each items. The mean scores of the FACT-G and FACT-AC component at 1^st^ measurement was 68.9 (±13.9) and 37.5 (±13.9), respectively. The test-retest reliabilities of each domain were adequate (intraclass correlation coefficient range, 0.601–0.825). Internal consistency of 17-items of the FACT-AC component was assessed. Cronbach’s α indicated “good” internal consistency at the level of 0.802 in the FACT-AC component, and ranged 0.776 to 0.818 if one item was deleted (Table 1). Results of linear mixed model for repeated measurement of Korean FACT-VCI showed that main effects of measurement time (1^st^ or 2^nd^) on AC component score were not significant (p = 0.589), while diversion type (ONB or IC) had trace effects on the score but failed to reach significance (p = 0.095; Table 2). The findings imply the absolute agreement between repeated tests of FACT-VCI.

**Table 1:** Korean version of the Functional Assessment of Cancer Therapy (FACT)-Vanderbilt Cystectomy Index (VCI) item variance with its test-retest reliability and internal consistency.

Domain or items	1^st^ measurement (N = 108)		2^nd^ measurement (N = 95)		Test-retest reliability*	Internal consistency**
	Mean	SD	Mean	SD		
FACT-G (37 items)						
Physical well-being domain (FACT-GP)	22.0	4.8	22.9	4.0	0.601 (0.401–0.734)	–
Social/family well-being domain (FACT-GS)	15.7	5.4	14.3	4.7	0.810 (0.715–0.874)	–
Emotional well-being domain (FACT-GE)	17.1	4.2	18.9	3.6	0.677 (0.514–0.785)	–
Functional well-being domain (FACT-GF)	15.1	6.1	13.3	6.4	0.825 (0.737–0.883)	–
Total sum	68.9	13.9	69.1	13.5	0.803 (0.704–0.869)	–
Additional concerns (FACT-AC) (17 items)						
Losing weight (C2)†	3.3	1.1	3.6	0.9	–	0.787
Bowel control (C3)	1.8	1.0	1.9	1.1	–	0.795
Diarrhea (C4)†	3.8	0.5	3.8	0.6	–	0.804
Good appetite (C6)	2.1	1.3	2.0	1.2	–	0.789
Content with appearance (C7)	1.9	1.1	1.7	1.1	–	0.787
Trouble controlling urine (BL1)†	2.8	1.2	2.8	1.2	–	0.783
Condition wakes me up at night (ITU7)†	2.5	1.2	2.9	1.0	–	0.778
Embarrassed my condition (ITU6)†	2.9	1.1	3.3	0.9	–	0.782
Caring for condition difficult (C9)†	2.8	1.0	3.1	1.0	–	0.780
Limit social interactions (ITU3)†	2.3	1.2	2.4	1.2	–	0.778
Limit physical activity (ITU4)†	2.3	1.2	2.4	1.3	–	0.776
Limit sexual activity (ITU5)†	1.6	1.6	1.5	1.5	–	0.806
Comfortable discussing with friends (ITU1)	1.4	1.2	1.3	1.2	–	0.807
Satisfied with urinary condition (VC1)	1.6	1.1	1.4	1.0	–	0.793
Afraid to go far from toilet (ITU2)†	3.0	1.1	3.1	1.1	–	0.798
Interested in sex (BL4)	1.0	1.3	1.1	1.3	–	0.818
Able to have erection (male only, BL3)	0.4	0.8	0.2	0.5	–	0.805
Total sum	37.5	8.9	38.2	8.2	0.822 (0.732–0.881)	0.802

SD, standard deviation; FACT-G, FACT-general; C, FACT-Colorectal; BL, FACT--Bladder Cancer; ITU, Functional Assessment of Incontinence Therapy-Urinary

*, the intraclass correlation coefficients with 95% confidence interval using samples of persons who completed 1^st^ and 2^nd^ measurement (N = 95), coefficient >0.6 is considered as indicative of good agreement

**, presented by Cronbach’s α of whole items and if item deleted, utilizing samples of persons who completed 1^st^ measurements (N = 108), α >0.7 is considered as indicative of internal consistency

^†^, item is reverse scored

**Table 2:** Differences in additional concerns (AC) component sum scores of Korean version of the Functional Assessment of Cancer Therapy (FACT)-Vanderbilt Cystectomy Index (VCI) scores by urinary diversion type with time.

	1^st^ measurement	2^nd^ measurement	Marginal Mean diversion
Orthotophic neobladder	36.00	37.64	36.74
Iieal conduit	38.88	38.68	38.76
Marginal Mean measurement	37.53	38.18	

Mean FACT-AC component sum scores were presented, higher sum scores indicated better health-related quality of life.

Main effect for measurement time, (1^st^ measurement vs. 2^nd^ measurement, 37.53 vs. 38.18; p = 0.589); main effect for diversion type, (orthotophic neobladder vs.ileal conduit, 37.64 vs. 38.68; p = 0.095); interaction between measurement time and diversion type, p = 0.320

### Principal components analysis for internal compositional validity assessment

Principal components analysis was performed to determine the internal compositional validity of 16 gender-neutral items of the FACT-AC component (Table 3). This model showed Bartlett’s test of sphericity (p <0.001) and Kaiser-Meyer-Olkin measure of sampling adequacy (0.718), indicating the adequacy of the model for factor analysis. Three components accounted for 49.3% of the total item covariance: “voiding problems”, “bowel problems”, “social/functional problems”. However, a single component solution accounted for only 27.6% of the total item covariance. All items except for C3 (diarrhea), ITU1 (comfortable discussing with friends), and BL4 (interest in sex) were substantial (varimax-rotated loading ≥0.3) with the one component solution.

**Table 3:** Principal components structure of data items for the additional concerns (AC) component of the Korean version of the Functional Assessment of Cancer Therapy (FACT)-Vanderbilt Cystectomy Index (VCI).

Items*	Three components**			Single component
	1	2	3	
Losing weight (C2)†	**0.634**			**0.601**
Bowel control (C3)		**0.340**	0.319	**0.366**
Diarrhea (C4)†	**0.578**		-0.330	0.192
Good appetite (C6)		**0.715**		**0.481**
Content with appearance (C7)		**0.699**		**0.413**
Trouble controlling urine (BL1)†	**0.726**			**0.693**
Condition wakes me up at night (ITU7)†	**0.660**			**0.723**
Embarrassed my condition (ITU6)†	**0.615**	0.319		**0.720**
Caring for condition difficult (C9)†	**0.585**	0.349		**0.734**
Limit social interactions (ITU3)†			**0.883**	**0.673**
Limit physical activity (ITU4)†			**0.879**	**0.669**
Limit sexual activity (ITU5)†			**0.465**	**0.304**
Comfortable discussing with friends (ITU1)		**0.490**		0.228
Satisfied with urinary condition (VC1)		**0.665**		**0.441**
Afraid to go far from toilet (ITU2)†	**0.579**			**0.429**
Interested in sex (BL4)	**-0.394**	0.340		-0.075
Proportion of covariance accounted for by component (initial eigenvalue)	19.5% (3.13)	15.4% (2.46)	14.4% (2.31)	27.6% (4.42)
Saturation (average loading) all 16 items	–	–	–	0.474
Coefficient alpha of the 16 item scale	–	–	–	0.718

C, FACT-Colorectal; BL, Fact-Bladder Cancer; ITU, Functional Assessment of Incontinence Therapy-Urinary

*, 16 gender-neutral items of FACT-AC component were included this principal components analysis, as one item (BL3, able to have erection) was gender-specific.

**, Entries are varimax-rotated loadings ≥0.3. These loadings indicate the strength of the association between a component and item. Bold values reflect the items with which the component is most strongly associated.

^†^, item is reverse scored

### Criterion-related validity in relation to the SF-36

Criterion-related validities of Korean FACT-VCI domains were also performed in relation to the SF-36 domain as follows: physical functioning (PF), role-functioning physical (RP), body pain (BP), general health (GH), vitality (VT), social functioning (SF), role-functioning emotional (RE), mental health (MH) domains, physical component summary (PCS), and mental health component summary (MCS). All domains of the FACT-G and FACT-AC components were significantly associated with related domains of the SF-36 (Pearson’s correlation coefficients range: 0.286–0.688; all p <0.01), except for associations between GS domain of the FACT-G component and related SF-36 domains (p = 0.775 and 0.057, respectively) and the FACT-AC component (p = 0.105; Table 4). Generally, the Korean FACT-VCI correlated well with the SF-36.

**Table 4:** Criterion-related validity of the Korean version of the Functional Assessment of Cancer Therapy (FACT)-Vanderbilt Cystectomy Index (VCI) in relation to the Short-Form 36-Item Health Survey (SF-36) version 2.0†.

	SF-36										FACT-G			
	PF	RP	BP	GH	VT	SF	RE	MH	PCS	MCS	GP	GS	GE	GF
FACT-G														
Physical well-being domain (GP)	0.286**	0.513**	0.503**	0.528**	0.460**	–	–	–	0.420**	–	–	–	–	–
Social/family well-being domain (GS)	–	–	–	–	–	0.028	–	–	–	0.184	–	–	–	–
Emotional well-being domain (GE)	–	–	–	–	–	–	0.350**	0.526**	–	0.442**	–	–	–	–
Functional well-being domain (GF)	–-	0.497**	–	–	–	–	0.422**	–	0.427**	0.547**	–	–	–	–
FACT-AC	0.384**	0.601**	0.469**	0.680**	0.650**	0.587**	0.534**	0.681**	0.525**	0.688**	0.589**	0.157	0.464**	0.615**

PF, physical functioning domain; RP, role-functioning physical domain; BP, body pain domain; GH, general health domain; VT, vitality domain; SF, social functioning domain; RE, role-functioning emotional domain; MH, mental health domain; PCS, physical component summary; MCS, mental health component summary; FACT-G, FACT-General; FACT-AC, FACT-Additional Concerns for Radical Cystectomy;

^†^, presented by Pearson’s correlation, it is generally considered that association is negligible at 0–0.3, with low positive at 0.3–0.5, moderate positive at 0.5-.7, and high positive at 0.7–1.0;

**, p <0.01;

*, p <0.05

## Discussion

Although some other tools for HRQOL assessment in bladder cancer have been developed, such as the FACT-Bladder Cancer (FACT-BL) [24], European Organization for Research and Treatment of Cancer (EORTC) QLQ-BLS, EORTC QLQ-BLM [25], or the Bladder Cancer Index (BCI) [26], only the BCI has psychometric validation [26]. The reliability and validity of original English version of FACT-VCI has been established [10,11].

### Reliability of original and Korean FACT-VCI

The test-retest reliability of FACT-VCI was first assessed by Cookson et al. [10]. The intraclass correlation coefficient of the AC component sum of the original FACT-VCI was 0.79, which indicated acceptable test-retest reliability [10]. A more recent psychometric validation study reported a higher correlation rate (ρ = 0.89; p <0.001) [11]. However, this higher rate may have been caused by the use of a different statistical methodology for the test-retest reliability assessment (Spearman correlation) [11]. Presently, we used similar methods as Cookson et al. [10] and demonstrated similar results (intraclass correlation coefficient = 0.822; Table 1). Therefore, the Korean FACT-VCI is concluded to have adequate test-retest reliability. Moreover, items of the FACT-G comportment also demonstrated ‘good’ reliability (intraclass correlation coefficient = 0.803; Table 1).

For the evaluation of internal consistency of the included items, the prior two studies similarly estimated the Cronbach’s α and reported similar values (0.85 and 0.854) [10,11]. Our study also adopted the same methodology, and showed a slightly lower but comparable rate of 0.802 (Table 1). Moreover, that rates were constant (0.776–0.818) if one item was deleted (Table 1), in agreement with one of the prior studies [11]. Therefore, we conclude that Korean FACT-VCI also has internal consistency among each item of the questionnaire.

To test the absolute agreement between repeated tests of FACT-VCI, a linear mixed model with the measurement time and urinary diversion type fixed was performed. The main effect of measurement time on the FACT-AC component score was not significant (p = 0.589; Table 2), which was consistent with the same finding by Cookson et al. that the main effect of measurement time was not significant (p = 0.66) [10]. However, our results differed in two points. First, the mean values of the AC component sum (37.5 and 38.2; Table 1) were slightly lower than those reported by Cookson et al. (44.38 and 45.21) [10]. The discrepancy is likely mainly due to the difference of patient characteristics. Our cohorts comprised of Asian populations with a shorter post-operative period (median 7.3 months) than the prior cohorts (more than one year) [10]. Second, our data showed trend toward better HRQOL in patients with IC than in patients with ONB (AC component score, 38.76 vs. 36.74; p = 0.095; Table 2), while the previous study demonstrated a trend toward compromised HRQOL in the IC cohort, compared with the ONB cohort (AC component score, 40.91 vs. 47.45; p = 0.08) [10]. However, Anderson et al. [11] and other researchers [26,27] reported similar results with us; patients with IC had better HRQOL outcomes compared to patients with ONB. These interesting findings that seemingly contradict common sense might be because of that some voiding problem-related items, such as BL1 (trouble controlling urine), ITU7 (condition wakes me up at night), VC1 (satisfied with urinary condition), or ITU2 (afraid to go far from toilet), can exaggerate the deterioration of HRQOL in ONB cohorts. However, to confirm this suggestion, a larger prospective study is needed.

### Validity of original and Korean FACT-VCI

Principal components anal**y**sis results provide some interesting information regarding this questionnaire (Table 3). The three component model accumulatively accounted for 49.3% of total item covariance, and the rate was comparable with accumulative accounting rates (58.0%) of the three component models of Anderson et al. [11]. This prior model could not confirm the representative criteria of each components, perhaps because the model was made using patients in a preoperative setting [11]. However, our three components were “voiding problems”, “bowel problems”, and “social/functional problems” (Table 3). Moreover, these components provided equivalent explanation powers to the total score (19.5%, 15.4%, and 14.4%, respectively; Table 3), and cumulative explanation of its three components (49.3%) was higher than that of the single component model (27.6%). Therefore, we believe that the internal compositional validity of the Korean FACT-VCI is adequate.

In the results of criterion-related validities, FACT-AC component scores showed good associations with all domains of SF-36 and FACT-G component, except for the GS (social/familial well-being) domain (p = 0.105). The GS domain was not also associated with SF (social functioning) domain and MCS (mental health component summary) of SF-36 (p = 0.775 and 0.057, respectively; Table 4). Similar findings were previously observed in the original version of FACT-VCI, in which the FACT-AC component score was not associated with the GS domain score of FACT-G component [10]. Thus, the dissociation between the GS domain and other domains stem from inherent characteristics of GS domain items, rather than being due to mistranslation. However, since other domains were well correlated with each other, it can be concluded that the Korean FACT-VCI has external validity.

### Limitations

Our study has some limitations. First, a substantial portion of the recruited subjects (14 of 108 subjects, 13.0%) did not complete the 2^nd^ measurement. To ensure proper statistical power, we endeavored to include maximal numbers of patient with available data required for analysis [28]. Second, because our subjects were predominantly male (95 of 108 subjects, 88.0%), we could not conclude whether the Korean FACT-VCI is gender-neutral. Instead, we excluded gender-dependent item (BL3, able to have erection) from some analyses (Table 3). Lastly, our cohorts did not comprise other UD types, such as Kock or Indiana pouch procedure. Therefore, it is still unclear whether the Korean FACT-VCI is applicable for those patients.

## Conclusions

This prospective study confirms the reliability and validation of the Korean FACT-VCI. The Korean FACT-VCI demonstrates comparable test-retest reliability, internal consistency, and internal and external validity compared to the original English version. This validated tool could be widely utilized in HRQOL studies of Korean patients with bladder cancer.
